# Vaccination Can Prevent Severe Pulmonary Disease in COVID-19 Positive Patients: A Case-Control Study

**DOI:** 10.7759/cureus.45638

**Published:** 2023-09-20

**Authors:** Sreesupria Ravichandran, Keerthika Vijayakumar, Vishwajit G. V., Siva P. M.

**Affiliations:** 1 Community Medicine, Government ESIC Medical College, Coimbatore, IND; 2 Community Medicine, Coimbatore Medical College, Coimbatore, IND; 3 Community Medicine, Government Medical College, Tiruppur, IND

**Keywords:** vaccine effectiveness, pulmonary involvement, covaxin, covishield, covid-19

## Abstract

Background: The COVID-19 pandemic was a global health emergency, which brought lives to a standstill. To combat this deadly virus, two vaccines were deployed widely: COVISHIELD (ChAdOx1 nCoV-19) and COVAXIN (BBV152). These were approved based on the immunological response they elicit in standardized conditions; however, the real-life scenario after deployment was completely different. Only in such situations can the true effectiveness of vaccines be assessed. The primary objective was to assess the effectiveness (VE) of COVAXIN/COVISHIELD in preventing severe pulmonary disease in RT-PCR-positive COVID-19 patients greater than 18 years of age.

Materials and methods: A case-control study was conducted among 260 subjects aged above 18 years, positive for COVID-19 through RT-PCR. 130 cases and 130 controls were enrolled. Radiological findings were obtained and subjects with >50% lung involvement were considered as cases. Subjects were interviewed about their vaccination status. Odds ratio was calculated, and the adjusted odds ratio was estimated for vaccine effectiveness, using the formula (1-adjusted ODDS ratio)*100.

Results: The vaccine effectiveness for a single dose of vaccine was 55.2% (95% C.I. 11.0%-77.5%) and with two doses was 98.0% (95% C.I. 85.0%-99.7%). Hence two doses are highly effective than a single dose of vaccine in reducing lung involvement.

Conclusion: Two doses of vaccine are more effective than a single dose vaccine in reducing lung involvement. Since sporadic cases of COVID-19 still persist, it is important to emphasize the role of vaccination in preventing severe COVID-19 infections, particularly in the elderly and those with comorbidities.

## Introduction

The COVID-19 pandemic was a global health emergency, which had brought our lives to a standstill. As of July 2023, COVID-19 has affected over 768 million people around the globe, with nearly seven million deaths [1]. In India, over 44 million people have been affected, with over 531,903 deaths [2].

Widespread vaccination drives were launched in many countries to combat this deadly virus. The vaccination drive in India had a promising start on January 16, 2021, with a gradual fall thereafter. The reason for this fall could have been multifactorial- probably due to negligence about the vaccine, being afraid to get the vaccine, lack of information and awareness about the safety of the vaccine, and lack of production and supply of the suitable vaccine candidate suitable to local needs, vaccine hesitancy. Complacency, distrust, lack of communication, and fear of side effects had aggravated this problem [3].

In India, there were predominantly two vaccines widely deployed in the vaccination drive: COVISHIELD (ChAdOx1 nCoV- 19) vaccine is a recombinant, replication-deficient adenoviral vector ChAdOx1, containing the SARS-CoV-2 spike glycoprotein antigen, developed by the Serum Institute of India Pvt. Ltd., manufactured under technology transfer from Oxford/Astrazeneca, and India's indigenous BBV152, a whole-virion inactivated SARS-CoV-2 antigen (Strain: NIV-2020-770), manufactured by Bharat Biotech, developed in collaboration with the Indian Council of Medical Research (ICMR) - National Institute of Virology (NIV), commonly called COVAXIN [4,5]. These vaccines were approved based on the immunological response they elicit, as evaluated from clinical trials, where the testing conditions were standardized, in a specific population thus providing key points that were required for vaccine licensure. However, the real-world scenario after vaccine deployment was entirely different, and only in such conditions, can the true protective effect of vaccines be assessed.

Although significant developments have been made in terms of treatment and prevention of COVID-19, vulnerable members of the population such as the elderly and high-risk individuals are still susceptible [6]. Transmission of the virus may be blocked by vaccination of younger age groups, but overall mortality can be reduced only if vaccination confers direct protective effects in vulnerable groups [7]. Ruch et al. studied the use of CT-imaging as a predictor of severe disease (early death/ICU admission) in COVID-19 patients and patients with lung involvement >50% were more likely to develop early severe disease, making lung involvement of disease an important prognostic factor [8].

Case-control studies are generally used to assess the effectiveness of vaccines after their implementation in public health programs by using endpoints of diseases like lung involvement [9]. Hence, post-vaccination evaluations are important means of providing information about prevention of disease endpoints like lung involvement and mortality. The main objective of our study is to estimate the vaccine effectiveness (VE) of BBV152 (COVAXIN)/ChAdOx1 nCoV-19 Vaccine (COVISHIELD) in reducing the severity of pulmonary involvement in patients with COVID-19 infection.

## Materials and methods

This is a hospital-based, single-center, retrospective case-control study conducted at a tertiary care center in Tamil Nadu with the data obtained from August 2022 to January 2023. The study population included hospitalized COVID-19 cases, confirmed by RT-PCR, above 18 years of age, with CT scans taken in the Radiology Department.

The cases were “people aged above 18 years who are RT-PCR positive for COVID-19 virus with severe pulmonary involvement (Lung involvement greater than 50%).” The controls were “people aged above 18 years who are RT-PCR positive for COVID-19 virus with mild pulmonary involvement (Lung involvement less than or equal to 50%, including nil Lung involvement).”

Inclusion criteria

Patients who were willing to participate in the study, who had been tested RT-PCR positive for COVID-19 infection with CT-chest taken.

Exclusion criteria

Pre-existing lung diseases, including previous COVID-19 infection, patients with inconclusive CT findings, and critically ill patients who were not able to provide details. Considering the vaccination status in cases as 40% and controls as 60%, with a 95% confidence interval and 80% power, the sample size was estimated as 100 cases and 100 controls. The case-control ratio was taken as 1:1. Total of 130 cases and 130 controls were enrolled, and all were included in the analysis.

Procedure

After getting clearance from the Institutional Ethical Committee (EC Reg No. ECR/892/Inst/TN/2016; Ref no: 0579/2021) and approval from the Department of Radiology, the RT-PCR-positive COVID-19 cases were enrolled. Data were collected by consecutive sampling. Study subjects were chosen from the list of COVID-19-positive patients provided by the Radiology Department. Consent was obtained from each participant. CT scan report of each participant was collected from the radiology department, and they were classified as cases (>50% lung involvement) and controls (<=50% lung involvement). Individual subjects were contacted to collect details regarding their demographics, vaccination status, and comorbidities (Diabetes, Hypertension, Coronary Artery Disease, Chronic kidney disease, and Chronic liver disease). The data collected were kept confidential.

Statistical analysis

Data were collected and entered in MS Excel, and analysis was done using the SPSS 21.0 version (IBM Corp., Armonk, NY). A chi-square test was done to find out the association between categorical variables. The crude ODDS ratio was calculated using univariate crosstabulation, and the adjusted ODDS ratio was calculated using Multiple logistic regression after adjusting with covariates (Age > 50 years, comorbidity, occupation status, and vaccine doses) and then VE was estimated from adjusted ODDS ratio, using the formula (1-adjusted ODDS ratio)*100. A p-value of less than 0.05 was considered significant. A p-value of less than 0.20 was included in the logistic regression analysis, performed using the “Enter Method.” Recall bias may have been present, but the data collected was cross-checked with the vaccine certificate of each individual to ensure reliability.

## Results

As seen in Table 1, most of the subjects were in the age group of above 50 years (cases - 67.7% vs controls - 60.0%), male gender (cases - 50.8% vs controls - 51.5%), and urban background (cases - 70.8% vs controls - 70.8%). Most of the patients were unemployed (cases - 50.0% vs controls - 49.2%) since the predominant age group was above 50 years. Comorbidities were present in 65.4% of cases and 56.9% of controls. Regarding the hospital admission status, 83.8% of cases and 86.2% of controls were admitted in wards with oxygen support. Only 16.2% vs 13.8% were admitted in intensive care units. There was no significant difference noted between any of the above-mentioned factors with cases and controls.

**Table 1:** Distribution of study subjects according to their socio-demographic profile among cases and controls NA - Not Applicable

Variables		Cases		Controls		Chi-square test	
		N	%	N	%	ODDS ratio (95% C.I.)	P-value
Age group	>50 years	88	67.7%	78	60.0%	1.397 (0.840 – 2.322)	0.197
	< =50 years	42	32.3%	52	40.0%		
Gender	Male	66	50.8%	67	51.5%	0.970 (0.596 – 1.577)	0.901
	Female	64	49.2%	63	48.5%		
Occupation	Semiprofessional	2	1.5%	9	6.9%	NA	0.064
	Skilled	26	20.0%	34	26.2%		
	Semiskilled	13	10.0%	9	6.9%		
	Unskilled	24	18.5%	14	10.8%		
	Unemployed	65	50.0%	64	49.2%		
Residence	Rural	38	29.2%	38	29.2%	1.000 (0.586 – 1.707)	1.000
	Urban	92	70.8%	92	70.8%		
Comorbidity	Present	85	65.4%	74	56.9%	1.429 (0.866 – 2.359)	0.162
	Absent	45	34.6%	56	43.1%		
Place of admission	ICU	21	16.2%	18	13.8%	1.199 (0.606 – 2.372)	0.602
	Ward	109	83.8%	112	86.2%		

Out of the total 130 cases only 19 (14.6%) received the COVID-19 vaccine and out of 130 controls 59 (45.4%) received the COVID-19 vaccine, as seen in Table 2. The crude odds ratio was 0.206 (95% C.I. 0.113%-0.374%, p<0.001). Regarding the type of vaccine, out of 19 vaccinated cases, all of them took the Covishield vaccine and in 59 vaccinated controls 53 received Covishield and six received Covaxin.

**Table 2:** Association between vaccination status and lung involvement NA - Not Applicable, Ref - Reference

Vaccination status	Cases		Controls		Chi-square test	
	N	%	N	%	ODDS ratio (95% C.I.)	P-value
Vaccinated	19	14.6	59	45.4	0.206 (0.113 – 0.374)	<0.001
Not vaccinated	111	85.4	71	54.6		
Total	130	100.0	130	100.0		
Covishield	19	14.6	53	40.8	0.229 (0.125 – 0.419)	<0.001
Covaxin	0	0.0	6	4.6	NA	0.003
Not Vaccinated	111	85.4	71	54.6	Ref	-
Total	130	100.0	130	100.0	-	-

Regarding the number of doses of vaccine received, out of 19 vaccinated cases 18 had only a single dose and only one patient received two doses of vaccine. Out of 59 vaccinated controls 28 received a single dose and 31 received two doses. The crude ODDS ratio for a single dose compared to unvaccinated was 0.41 (95% C.I. 0.21 - 0.80, p=0.008), and for two doses 0.02 (95% C.I. 0.003%-0.155%, p<0.001), which is shown in Table 3.

**Table 3:** Association between number of doses and lung involvement Ref - Reference

Number of doses	Cases		Controls		Chi-square test	
	N	%	N	%	ODDS ratio (95% C.I.)	P-value
Two doses	1	0.8	31	23.8	0.021 (0.003 – 0.155)	<0.001
One dose	18	13.8	28	21.5	0.411 (0.212 – 0.798)	0.008
Not Vaccinated	111	85.4	71	54.6	Ref	-
Total	130	100.0	130	100.0	-	-

To estimate the VE, covariates with a p-value<0.20 for crude OR were selected for multiple logistic regression to get the adjusted odds ratio. The covariates included in the logistic regression analysis were age group, occupation, and comorbid status, and along with that vaccination status was included. After doing regression analysis the adjusted ODDS ratio was estimated as 0.214 (95% C.I. 0.116%-0.396%, p<0.001), as seen in Table 4. The VE ((1-adjusted ODDS ratio)*100) was estimated to be 78.6% (95% C.I. 60.4%-88.4%), which shows that vaccination has a protective effect over severe lung involvement.

**Table 4:** Multiple logistic regression to estimate the effectiveness of vaccination B - Beta Coefficient, S.E - Standard Error, Sig - Significance, Exp(B) - Adjusted odds ratio, C.I. - Confidence Interval

Variables	B	S.E.	Sig.	Exp(B)	95% C.I.for EXP(B)	
					Lower	Upper
Age >50 years	0.297	0.298	0.319	1.346	0.750	2.416
Presence of any comorbidity	0.109	0.282	0.700	1.115	0.642	1.935
Occupation - Unemployed	-	-	0.179	-	-	-
Semiprofessional	-1.169	0.848	0.168	0.311	0.059	1.637
Skilled	-0.132	0.349	0.706	0.877	0.443	1.737
Semiskilled	0.283	0.510	0.579	1.327	0.489	3.603
Unskilled	0.716	0.414	0.084	2.046	0.908	4.609
Vaccinated	-1.542	o.314	<0.001	0.214	0.116	0.396
Constant	0.122	0.364	0.738	1.130	-	-

While doing regression analysis with vaccination dosage the adjusted ODDS ratio for a single dose was 0.448 (95% C.I. 0.225%-0.890%, p=0.022), and for two doses was 0.020 (95% C.I. 0.003%-0.150%, p<0.001), as seen in Table 5. So, the effectiveness of a single dose of vaccine was 55.2% (95% C.I. 11.0%-77.5%), and with two doses was 98.0% (95% C.I. 85.0%-99.7%).

**Table 5:** Multiple logistic regression to estimate the effectiveness of number of doses of vaccine B - Beta Coefficient, S.E - Standard Error, Sig - Significance, Exp(B) - Adjusted odds ratio, C.I. - Confidence Interval

Variables	B	S.E.	Sig.	Exp(B)	95% C.I.for EXP(B)	
					Lower	Upper
Age >50 years	0.313	0.311	0.315	1.367	0.743	2.515
Presence of any comorbidity	0.196	0.287	0.493	1.217	0.694	2.134
Unemployed	-	-	0.148	-	-	-
Semiprofessional	-1.243	0.843	0.140	0.289	0.055	1.505
Skilled	-0.097	0.359	0.786	0.907	0.449	1.833
Semiskilled	0.285	0.515	0.580	1.330	0.485	3.648
Unskilled	0.803	0.443	0.070	2.232	0.937	5.317
Vaccine Doses – Not Vaccinated	-	-	0.000	-	-	-
Single dose	-0.803	0.351	0.022	0.448	0.225	0.890
Two doses	-3.927	1.036	<0.001	0.020	0.003	0.150
Constant	0.039	0.372	0.915	1.040	-	-

## Discussion

Vulnerable populations have been the worst affected by the COVID-19 pandemic, which includes people living in poverty, the elderly, people with disabilities, and racial and ethnic minorities [10]. Despite WHO declaring the end of the pandemic, members of these groups continue to suffer from the risk of COVID-19-related mortality and morbidity. As stressed by the WHO, it is important to transition to the long-term management of the COVID-19 infection, where vaccinations will continue to play a vital role [11].

Li et al. have found that there was no significant association between age distribution and the group that had more severe CT changes (p = 0.074) [12]. Similarly, in our study, we found no significant difference in the age distribution of cases and controls (67.7% of cases and 60% of the controls were greater than 50 years old). This further supports the notion that age may not be a determining factor in the observed changes.

In the same study [12], male gender was significantly associated with the group that had higher lung consolidation on CT (p=0.019). But our study showed no significant difference in gender (p=0.901). Comorbidities were present in 65.4% of cases and 56.9% of controls, with hypertension and diabetes being the most common, and the severity of disease is higher among this group of patients. This is similar to a study by Sharma et al. [13], where comorbidities were present in 28.6% of people, with hypertension and diabetes being the most common and these individuals presented with increased disease incidence and severity.

In our study, 14.6% of cases (severe lung involvement) and 45.4% (mild lung involvement) of controls had received at least a single dose. The crude odds ratio of 0.206 indicates the protective effect of vaccination against severe lung involvement among controls. A previous study by Lee et al. has explored in detail the imaging and clinical features of COVID-19 breakthrough infections, which also showed that vaccinated patients had a lower risk of COVID-19 pneumonia and intensive care unit admission [14].

Guan et al. said that interstitial abnormalities seen on CT on admission were significantly correlated with the patients' endpoints, such as discharge, intensive care unit admission, and mechanical ventilation [15]. These findings were supported by Ruch et al., who found that >50% of lung involvement was associated with early severe disease (ICU admission/death). Hence, by vaccination, the survival outcome of COVID-19 patients can be improved, by preventing ICU admission and death [8].

The overall VE in our study was estimated at 78.6%. The effectiveness of a single dose of vaccine was 55.2% and with two doses was 98.0%. This is in concordance with the meta-analysis by Zhang et al., which showed that multiple vaccinations in the elderly proved to be more effective. This also supported the use of booster doses to improve immunity [16].

With the emergence of different variants of the virus, such as the delta and omicron variants, there is a high possibility of a drop in vaccine efficacy. Paul et al. conducted a case-control study in Scotland, where the efficacy of the ChAdOx1 vaccine against severe Delta variant COVID-19 waned substantially in 20 weeks from the second dose. The emergence of the Delta variant caused a temporary increase in relative risk in patients who had only received a single dose, while the relative risk remained unchanged for those who had received two doses [17]. Andrews et al. showed that double-dose vaccination with ChAdOx1 nCoV-19 conferred no protection against the Omicron variant 20 weeks post-vaccination [18]. A study conducted in AIIMS, Bhuvaneshwar, found COVAXIN (BBV152) to have low efficacy against preventing breakthrough infections [19]. While the above studies have analyzed the vaccine's ability to prevent infections, they have not looked into its ability to prevent severe lung involvement as a specific endpoint. But a study by ICMR shows that BBV152 has 69% efficacy in preventing severe infections. By using severe pulmonary involvement (lung involvement > 50%) as an outcome measure, we have kept ascertainment bias to the minimum. The efficacy of the vaccine against hospitalization may be underestimated if its efficacy against test-positive infection is lower than the efficacy against the disease [8]. Hence, in our case-control study, we have only evaluated the efficacy of the vaccine against severe pulmonary involvement among hospitalized patients.

A limitation of our study is that we have considered both Covishield and Covaxin under the same umbrella of vaccination and have not made a distinction in their efficacy. Moreover, we did not perform genomic sequencing of the virus to accurately determine the infective strain. When the study was being conducted, both the Omicron and Delta variant were predominant in Tamil Nadu. A study by Wong et al. stated that the maximum severity of chest radiographic changes peaked at around six to 11 days from symptom onset. As the CT in our hospital was taken immediately on admission, this time frame of six to 11 days from symptom onset might not have coincided [20].

## Conclusions

The overall efficacy of vaccination against COVID-19 is 78.6%, using the severity of lung involvement as the primary outcome. Preventing severe lung involvement can significantly reduce morbidity and mortality by decreasing the need for oxygen supplementation, ICU admission, and the risk of early death. Two doses of vaccination significantly improve efficacy compared to one, and hence, all people, especially vulnerable groups like the elderly should be urged to complete their double dose of vaccination. However, more research has to be done on waning immunity after two doses of vaccination, thus examining the importance of booster doses.

In conclusion, our study highlights the impact of the COVID-19 pandemic on vulnerable populations, emphasizing the critical role of vaccinations in its long-term management. It is imperative that we remain vigilant and adaptable in our strategies, as new variants of COVID-19, including recently identified ones like EG.5 (Eris), continue to emerge. By drawing lessons from the past years, we can protect ourselves against these evolving threats.
